# THROUGH THE EYES OF CARE MANAGERS: UNDERSTANDING THE VALUE OF MEMBERSHIP TO THE AGING LIFE CARE ASSOCIATION

**DOI:** 10.1093/geroni/igae098.1041

**Published:** 2024-12-31

**Authors:** Carlisle Shealy, Pamela Teaster, Jullie Gray, Laura Sands

**Affiliations:** Virginia Tech Center for Gerontology, Blacksburg, Virginia, United States; Virginia Tech., BLACKSBURG, Virginia, United States; University of Washington, Seattle, Washington, United States; Virginia Tech, Blacksburg, Virginia, United States

## Abstract

Care managers, also known as Aging Life Care Professionals (ALCPs), have specialized knowledge pertaining to aging and disability. Through care coordination, they serve a critical role for families, caregivers, and older adults themselves. The Aging Life Care Association (ALCA) is a nonprofit member association aimed at promoting the work of ALCPs by offering continuing education, professional development, and ethical standards. To fill a gap in understanding the organization, we explored the usefulness and benefits of ALCA membership. We conducted 35 individual virtual interviews with members, exploring satisfaction with membership, organizational support to members, types of assistance members received, and engagement activities. Chapter presidents were recruited first, and afterwards, we used snowball sampling to recruit diverse members. Interviews were transcribed and thematically coded. Care managers joined ALCA for numerous reasons including networking, connections, and camaraderie. Relationships with other members contributed to why members remained in the organization. Care managers sought an association that gave their work professional credibility and standards. Members valued referrals, resources for growing a business, online educational resources online, and conferences. Participants suggested opportunities for greater inclusion of new members, especially those from diverse racial and ethnic and racial backgrounds as well as how to best apply ALCA’s Code of Ethics and Standards of Practice. Others wanted education tailored for different career stages. Although opportunities exist to address challenges, membership in ALCA can improve access to information and services needed to coordinate improved care for older adults while also enhancing quality of care.

